# Biopsychosocial pain assessment and management in paediatric inflammatory vs non-inflammatory musculoskeletal conditions: a vignette study

**DOI:** 10.1093/rap/rkag007

**Published:** 2026-01-19

**Authors:** Danielle C Mountain, Daniela Ghio, Lis Cordingley, Janet E McDonagh, Sarah Peters, Rebecca R Lee

**Affiliations:** Division of Musculoskeletal and Dermatological Sciences, Faculty of Biology, Medicine and Health, Centre for Epidemiology Versus Arthritis, Centre for Musculoskeletal Research, University of Manchester, Manchester Academic Health Science Centre, Manchester, UK; National Institute for Health Research Biomedical Research Centre, Manchester University Hospital NHS Trust, Manchester, UK; Division of Psychology and Mental Health, Manchester Centre for Health Psychology, University of Manchester, Manchester, UK; Faculty of Health, Social Care and Medicine, Edge Hill University, Ormskirk, UK; Division of Psychology and Mental Health, Manchester Centre for Health Psychology, University of Manchester, Manchester, UK; Division of Musculoskeletal and Dermatological Sciences, Faculty of Biology, Medicine and Health, Centre for Epidemiology Versus Arthritis, Centre for Musculoskeletal Research, University of Manchester, Manchester Academic Health Science Centre, Manchester, UK; National Institute for Health Research Biomedical Research Centre, Manchester University Hospital NHS Trust, Manchester, UK; Division of Psychology and Mental Health, Manchester Centre for Health Psychology, University of Manchester, Manchester, UK; Division of Musculoskeletal and Dermatological Sciences, Faculty of Biology, Medicine and Health, Centre for Epidemiology Versus Arthritis, Centre for Musculoskeletal Research, University of Manchester, Manchester Academic Health Science Centre, Manchester, UK; National Institute for Health Research Biomedical Research Centre, Manchester University Hospital NHS Trust, Manchester, UK; Division of Psychology and Mental Health, Manchester Centre for Health Psychology, University of Manchester, Manchester, UK; Division of Musculoskeletal and Dermatological Sciences, Faculty of Biology, Medicine and Health, Centre for Epidemiology Versus Arthritis, Centre for Musculoskeletal Research, University of Manchester, Manchester Academic Health Science Centre, Manchester, UK; National Institute for Health Research Biomedical Research Centre, Manchester University Hospital NHS Trust, Manchester, UK; Division of Psychology and Mental Health, Manchester Centre for Health Psychology, University of Manchester, Manchester, UK

**Keywords:** chronic pain, paediatric rheumatology, children, adolescent, pain assessment, pain management

## Abstract

**Objectives:**

There is no research evidence about how healthcare professionals (HCPs) prioritise assessment and management of pain in different paediatric chronic musculoskeletal conditions (e.g. inflammatory or non-inflammatory). This study investigated and compared paediatric rheumatology HCPs’ pain assessment/management priorities in inflammatory and non-inflammatory chronic musculoskeletal conditions and explored perceived barriers to implementation of prioritised pain assessment/management approaches.

**Methods:**

Participants were presented with online vignettes describing a young person with an inflammatory (JIA) or non-inflammatory (diffuse idiopathic pain) chronic musculoskeletal condition. Participants completed closed questions on pain assessment/management priorities and open questions on perceived barriers to implementation of these priorities in clinical practice. Data were analysed using within-subjects bivariate statistical analysis and content analysis.

**Results:**

Results from 56 HCPs (11 countries) found that broadly similar pain assessments were selected for both conditions. Biomedical management approaches were more frequently selected for the inflammatory condition, whereas psychosocial approaches were primarily selected for the non-inflammatory condition. Barriers to implementation of assessment/management approaches included limited time, resources, knowledge and skillset and HCPs’ habits and beliefs about pain care (e.g. limiting access to components of biopsychosocial pain management due to preconceived beliefs about the musculoskeletal condition).

**Conclusion:**

Paediatric rheumatology HCPs generally prioritise similar pain assessments for inflammatory and non-inflammatory chronic musculoskeletal conditions. However, some HCPs perceive psychosocial pain management approaches as less important for managing pain in inflammatory conditions. Importantly, pain is always biopsychosocial in nature and clinical guidelines (that emphasise the biopsychosocial perspective) should ideally be followed regardless of condition type.

Key messages

- Some paediatric rheumatology healthcare professionals do not implement biopsychosocial pain management for chronic musculoskeletal conditions.
- Limited time, resources and knowledge/skillsets and personal habits and beliefs impede provision of biopsychosocial care.
- Future research should explore education on pain causes to reconceptualise healthcare professionals’ understanding of pain.

## Introduction

Paediatric chronic musculoskeletal pain conditions are a global economic and health concern that can considerably impact children’s and young people’s (CYP’s) physical, psychological and social functioning and persist into adulthood [1–4]. Chronic musculoskeletal pain (i.e. persistent or episodic pain for >3 months) can be a primary condition (i.e. a disease) or secondary to an underlying condition (e.g. inflammation associated with JIA) [5, 6]. A relevant categorisation used within paediatric rheumatology settings, where these conditions are predominantly referred and managed, is whether pain occurs in the context of an inflammatory or non-inflammatory musculoskeletal condition [7–10]. This categorisation captures differences in disease processes between different pain types. However, it risks oversimplifying the complex nature of chronic pain experiences that are influenced by biological, psychological and social (biopsychosocial) contributing factors. CYP can experience components of one or both types of pain (i.e. inflammatory and/or non-inflammatory) that can evolve and transition over time [7, 11, 12].

Given this impact on CYP’s functioning, effective and timely assessment and management of pain is crucial. It is important for healthcare professionals (HCPs) to direct attention to biological, psychological and social contributing factors for inflammatory or non-inflammatory conditions [13]. However, emerging evidence from studies of healthcare communication in paediatric rheumatology suggests there may be differences in HCPs’ beliefs and decisions when managing pain for inflammatory versus non-inflammatory conditions [10, 14]. Some HCPs focus only on assessing and treating identifiable physiological markers of disease that they associate with pain (e.g. disease activity) for individuals with JIA, an inflammatory condition [10]. This occurs despite evidence that pain can persist when pharmacological drugs have controlled inflammation [15] and despite clinical recommendations to assess and manage both disease and pain [1, 16]. In contrast, comprehensive psychosocial pain management is often viewed as only relevant for CYP experiencing non-inflammatory pain conditions [10].

Dichotomising assessment and treatment approaches in this way can mean that some CYP are not exposed to current best practices in pain management, which limits their ability to self-manage their own pain, both current and future episodes. Integrated biopsychosocial management of pain is guided by comprehensive pain assessments, such as history taking, physical examination and age-appropriate psychometric tools [1, 13, 17]. Tailoring pain management to the individual child and combining relevant pharmacological (e.g. analgesics), physical (e.g. physical therapy), psychological [e.g. cognitive behavioural therapy (CBT)] and social (e.g. family therapy) components can provide CYP with crucial pain self-management skills to improve long-term health outcomes (e.g. anxiety, perceived physical functioning) [13, 17–21].

Although healthcare communication studies suggest CYP’s pain may be managed differently to clinical recommendations, no research has directly explored this. It is unclear which assessment (e.g. imaging, CYP’s behaviours) and management approaches (e.g. talking therapies, physical therapies) HCPs differentially prioritise between musculoskeletal conditions (inflammatory or non-inflammatory). It is important to examine these differences, as HCPs’ pain beliefs influence CYP’s pain beliefs, coping methods and health and pain outcomes [22, 23]. CYP can perceive that their pain is dismissed or invalidated by HCPs if professionals cannot identify a cause for their pain (e.g. in non-inflammatory conditions) or their pain occurs despite disease activity being well-controlled (e.g. in inflammatory conditions) [10, 24]. Experiences of dismissal and invalidation increase patient’s hostility towards professionals and reduces treatment adherence [25].

While biopsychosocial pain assessment and management is the gold standard, there are significant barriers to their real-world provision. Known barriers for paediatric chronic pain conditions include HCPs’ confidence (e.g. in managing chronic pain) and limited time, service availability (e.g. pain services) and training in paediatric pain [9, 10, 17, 26, 27]. No research has explored whether HCPs face similar or differing barriers to the provision of perceived best clinical practice for CYP with inflammatory or non-inflammatory musculoskeletal conditions. It is important to identify where care needs to be improved to facilitate the implementation of best practice approaches and ultimately improve CYP’s health outcomes.

The aim of the study was to describe and compare paediatric rheumatology HCPs’ decisions about pain assessment and management priorities between paediatric chronic musculoskeletal conditions (inflammatory or non-inflammatory). A secondary aim was to investigate and compare barriers to implementation of these priorities in clinical practice between conditions.

## Methods

### Study design

This was a cross-sectional questionnaire study delivered via Qualtrics (Provo, UT, USA; November 2021), an online questionnaire delivery system. The questionnaire employed close-ended and open-ended items in relation to fictional clinical vignettes. The Strengthening the Reporting of Observational Studies in Epidemiology for cross-sectional studies was followed for reporting purposes (Supplementary Table S1) [28]. Ethical approval was obtained by the University of Manchester Research Ethics Committee (ref: 2021-11480-21036).

### Participants

Participants were HCPs working within paediatric and/or adolescent rheumatology centres internationally. Participants were eligible if they were involved in and had experience making clinical management decisions for CYP with chronic musculoskeletal conditions (no minimum number of years of experience required) and were fluent in English. This included consultant paediatric/adolescent rheumatologists (and those in training), consultant adult rheumatologists who see adolescents, general paediatricians with an interest in paediatric rheumatology, occupational therapists, physiotherapists, nurses, psychologists, doctors undergoing specialist paediatric training and pain specialist doctors. Participants were recruited through a larger observational questionnaire (Mountain *et al*., manuscript in preparation) that explored HCPs’ beliefs about pain experiences. A minimum sample size of 34 participants was deemed suitable from this larger questionnaire to assess within-subject comparisons and considering the limited number of HCPs in paediatric rheumatology settings.

Participants were recruited between November 2021 to July 2022. Convenience and snowball sampling strategies were employed by distributing advertisements via social media (Twitter, Facebook), a professional e-mail list server (PEDIATRIC-PAIN) and through hosts of professional clinical organisations, including the Barbara Ansell National Network for Adolescent Rheumatology [29], the British Society for Rheumatology and the Paediatric Rheumatology Clinical Studies Group [30]. Advertisements contained a weblink to Qualtrics and encouraged participants to distribute the questionnaire to other HCPs. Participants provided electronic informed consent on Qualtrics after reading the participant information sheet but prior to beginning the questionnaire.

### Vignettes

Vignettes are short, descriptive scenarios that can be utilised to explore HCP decision-making processes in the context of real-world situations [31]. Vignettes are useful for exploring decision-making by manipulating variables within the vignette (e.g. the type of condition) to explore how these influence decisions while controlling for potentially confounding variables/factors in real-world contexts (e.g. the patient’s behaviour) [31].

Fictional vignettes were created through expert panel consensus within the team, who have extensive experience in conducting clinical research in paediatric pain and rheumatic conditions. This included health psychologists (D.C.M., D.G., L.C., R.R.L.) and an adolescent rheumatologist (>20-years’ experience) who was experienced in managing chronic musculoskeletal pain conditions (inflammatory and non-inflammatory) in rheumatology clinics (J.E.M.D.). Three UK-based paediatric/adolescent rheumatology HCPs (two rheumatologists, one physiotherapist) piloted the vignettes. They provided feedback on the content’s comprehensibility and relatability, including whether enough clinical information was provided to make informed decisions. Based on this, brief descriptions of clinical presentations were added to aid judgements of inflammation (e.g. joint swelling versus allodynia).

The vignettes described a young person with a musculoskeletal condition (inflammatory, JIA/non-inflammatory, diffuse idiopathic pain) reporting chronic pain at a 6-month post-diagnosis appointment (Supplementary Data S1). Gender (male/female) was counterbalanced to control for potential gender effects in HCPs’ interpretations of the vignettes. All participants viewed both an inflammatory and non-inflammatory vignette, the order of which Qualtrics randomly assigned. Brief clinical observations aided participants’ perception of an inflammatory (swollen joint) or non-inflammatory (allodynia) condition. Minor changes to the vignette wording reduced the likelihood of participants noticing manipulations, such as changing the order of non-critical filler words.

### Pain assessment and management priorities questionnaire

The questionnaire (Supplementary Data S2) included a demographic section [gender, job title, country, years registered as a HCP and in the paediatric speciality, confidence in their understanding of pain (0 not at all confident, 100 confident) and percentage workload of patients with inflammatory conditions or non-inflammatory conditions with or without a known cause].

The authors developed the questionnaire to explore HCPs’ biopsychosocial pain assessment/management priorities. Priorities were chosen using recommendations in the paediatric chronic pain literature [1, 7, 18, 32], the International Association for the Study of Pain’s ‘Pain Curricula’ [33], a validated multidimensional pain assessment tool developed by the authors called My Pain Tracker [34, 35] and the teams’ extensive knowledge and experience in the clinical management of paediatric musculoskeletal conditions and chronic pain. The same three HCPs also piloted and informed changes, such as refining a pain assessment category from ‘interference (e.g. with daily activities)’ to ‘interference (e.g. daily activities, hobbies, sports)’.

Participants scored how important they believed each option was for understanding or helping the young person’s pain using a 5-point Likert-type response (‘not at all important’ to ‘very important’) and could select ‘Unfamiliar with method’ for pain management options. A closed question asked participants whether the options they perceived as ‘important’ or ‘very important’ would be conducted or recommended in real-world clinical practice (yes, no, maybe). An open question containing an essay text response box (no minimum or maximum word count) asked participants why they believed a method would not or might not be conducted or recommended.

### Data analysis

For the quantitative data, statistical analysis was conducted using SPSS 25.0 (IBM, Armonk, NY, USA) [36]. Descriptive statistics [e.g. median and interquartile range (IQR)] were calculated for demographics and beliefs of whether pain assessment or management priorities would be conducted/recommended in real-world clinical practice.

Pain assessment and management 5-point Likert-type responses were collapsed into binary variables to assess within-subject differences in priorities between vignettes. This approach is recommended for Likert-type adjective scales that do not have equal intervals and are not considered continuous [37]. ‘Very important’ and ‘important’ became ‘important’. ‘Not at all important’, ‘low importance’ and ‘neutral’ became ‘not important/neutral’. The McNemar test assessed differences in binary-paired variables. Multiple comparisons were controlled using Holm–Bonferonni correction [38, 39] and adjusted *P*-values were reported. Data were not included in pairwise analysis if participants were ‘unfamiliar’ with a pain management method. There were no missing quantitative outcome data (ratings of the importance of pain assessment/management approaches). One participant had missing demographic data (they did not report their patient workload). *P*-values <0.05 were deemed significant for all statistical tests.

Participants’ free-text responses about barriers to providing pain assessment and management in real-world clinical practice were subjected to manifest and latent content analysis [40] in NVivo 12 (Lumivero, Denver, CO, USA). Inflammatory and non-inflammatory responses were analysed separately for group-specific coding. Responses ranged from a couple of words to multiple sentences but were typically less than four sentences. One author (D.C.M.) familiarised herself with the dataset before inductively coding it by grouping words, phrases or sentences into codes relevant to the study aims. Related codes (e.g. ‘lack of training’ and ‘lack of knowledge’) were grouped into subcategories (e.g. ‘limited pain assessment and/or management knowledge and skills’). A working codebook was created by combining subcategories with shared latent meanings into overarching categories. A second author (D.G.) coded 25% of the data using the codebook. Interrater reliability between authors was moderate (k = 0.71) and increased (k = 0.79) following discussion of discrepancies [41]. The codebook was refined in discussions with all authors to ensure the categories appropriately reflected the content. One author (D.C.M.) analysed the remaining data, summarised findings for each condition and discussed similarities and differences across conditions with all authors in multiple meetings.

## Results

A total of 83 HCPs consented to take part, of which 27 partially completed the questionnaire (5 provided only consent, 8 completed/partially completed only the demographic section and 14 completed/partially completed only one vignette). Reasons for non-completion were not provided. Fifty-six (79% female) completed the questionnaire and were included in analysis (see Table 1). Most participants were physiotherapists (30%) or consultant paediatric/adolescent rheumatologists (21%) from 11 countries (54% UK, 13% USA). The median years registered as a HCP and within the paediatric rheumatology speciality were 16.5 years (IQR 15) and 11.0 years (IQR 14), respectively. Participants were mostly confident in their understanding of pain [median 79.5 of 100 (range 42–100)].

**Table 1.** HCPs’ demographic information (*N* = 56).

Demographic characteristics	Values
Female, *n* (%)	44 (78.6)
Years registered as HCP, median (IQR)	16.5 (69.3, 84.5)
Years registered in paediatrics, median (IQR)	11.0 (6.0, 20.0)
Confidence in their pain understanding (0, not at all confident; 100, confident), median (IQR)	79.5 (10.0, 25.0)
Job role, *n* (%)	
Consultant paediatric or adolescent rheumatologist	12 (21.4)
Consultant adult rheumatologist who sees adolescents	2 (3.6)
Paediatric or adolescent rheumatologist in training	2 (3.6)
General paediatrician	2 (3.6)
Occupational therapist	4 (7.1)
Physiotherapist	17 (30.4)
Nurse	9 (16.1)
Psychologist	1 (1.8)
Doctors undergoing specialist paediatric training	3 (5.4)
Pain specialist doctor (all grades)	3 (5.4)
Other^a^	1 (1.8)
Country, *n* (%)	
Australia	6 (10.7)
Canada	5 (8.9)
Turkey	2 (3.6)
UK	30 (53.6)
USA	7 (12.5)
Other	6 (10.8)
Percentage of patient workload—inflammatory^b^, *n* (%)	
<25%	12 (21.8)
25–50%	11 (20.0)
50–75%	19 (34.5)
>75%	13 (23.6)
Percentage of patient workload—non-inflammatory and a known cause^b^, *n* (%)	
<25%	26 (47.3)
25–50%	16 (29.1)
50–75%	11 (20.0)
>75%	2 (3.6)
Percentage of patient workload—non-inflammatory and no known cause^b^, *n* (%)	
<25%	35 (63.6)
25–50%	16 (29.1)
50–75%	2 (3.6)
>75%	2 (3.6)

a The ‘other’ job category referred to a community paediatrician.

b Missing data, *n* = 55. Countries with only one respondent were categorised as ‘other’.

### HCPs’ pain assessment and management priorities

Table 2 presents significant differences in HCPs’ pain assessment and management priorities for understanding or helping pain by condition. Most pain assessments were perceived as important for both conditions. However, two biomedical assessments (laboratory tests, imaging investigations) were perceived as important for the inflammatory condition but not the non-inflammatory condition. Two psychosocial assessments (history of psychosocial trauma, perception of parent/caregiver) were perceived as important for the non-inflammatory condition but not the inflammatory condition. All pain feature assessments were a priority irrespective of condition, except the number of pain locations, which was perceived as important for the inflammatory condition.

**Table 2:** Changes in perceived importance of pain assessment/management approaches between vignettes.

General pain assessments	Not important/neutral for both conditions, *n* (%)	Important for both conditions, *n* (%)	Important for only the inflammatory condition, *n* (%)	Important for only the non-inflammatory condition, *n* (%)	*P*-value
Physical examination	0 (0)	52 (92.9)	4 (7.1)	0 (0)	1.000
Family medical history	8 (14.3)	38 (67.9)	4 (7.1)	6 (10.7)	1.000
Medical history	2 (3.6)	48 (85.7)	6 (10.7)	0 (0)	0.651
History of physical trauma	1 (1.8)	46 (82.1)	3 (5.4)	6 (10.7)	1.000
Pain history	0 (0)	53 (94.6)	2 (3.6)	1 (1.8)	1.000
Sleep history	0 (0)	52 (92.9)	0 (0)	4 (7.1)	1.000
Laboratory tests	13 (23.2)	17 (30.4)	25 (44.6)	1 (1.8)	<0.001
Imaging investigations	16 (28.6)	14 (25.0)	26 (46.4)	0 (0)	<0.001
Pain interference	0 (0)	54 (96.4)	1 (1.8)	1 (1.8)	1.000
History of psychosocial trauma	0 (0)	41 (73.2)	0 (0)	15 (26.8)	0.002
How the young person thinks	0 (0)	53 (94.6)	0 (0)	3 (5.4)	1.000
Emotion	0 (0)	51 (91.1)	0 (0)	5 (8.9)	1.000
Pain behaviour	0 (0)	54 (96.4)	0 (0)	2 (3.6)	1.000
Perception of parent/caregiver	0 (0)	45 (80.4)	0 (0)	11 (19.6)	0.024
School experience(s)	0 (0)	51 (91.1)	0 (0)	5 (8.9)	1.000

Pain feature assessments	Not important/neutral for both conditions, *n* (%)	Important for both conditions, *n* (%)	Important for only the inflammatory condition, *n* (%)	Important for only the non-inflammatory condition, *n* (%)	*P*-value
Pain intensity	2 (3.6)	41 (73.2)	9 (16.1)	4 (7.1)	1.000
Location of pain	1 (1.8)	42 (80.4)	11 (19.6)	2 (3.6)	0.494
Number of pain locations	1 (1.8)	37 (66.1)	17 (30.4)	1 (1.8)	0.004
Extent of pain (e.g. spread)	3 (5.4)	44 (78.6)	5 (8.9)	4 (7.1)	1.000
Pain quality (e.g. ache, burn)	11 (19.6)	37 (66.1)	6 (10.7)	2 (3.6)	1.000
Pain time of day	0 (0)	47 (83.9)	8 (14.3)	1 (1.8)	0.742
Initial onset of pain	4 (7.1)	45 (80.3)	5 (89.3)	2 (3.6)	1.000
Frequency of occurrence of pain	0 (0)	51 (91.1)	3 (5.4)	2 (3.6)	1.000

Pain management approaches	Not important/neutral for both conditions, *n* (%)	Important for both conditions, *n* (%)	Important for only the inflammatory condition, *n* (%)	Important for only the non-inflammatory condition, *n* (%)	*P*-value
NSAIDs	5 (9.4)	6 (11.3)	42 (79.2)	0 (0)	<0.001
Anti-rheumatic drugs	9 (17.6)	2 (3.9)	40 (78.4)	0 (0)	<0.001
Analgesic drugs	36 (67.9)	3 (5.7)	10 (89)	4 (7.5)	1.000
Other drugs (e.g. antidepressants)	28 (56.0)	2 (4.0)	1 (2.0)	19 (38.0)	0.001
Topical agents	15 (27.8)	23 (42.6)	9 (16.7)	7 (13.0)	1.000
Other non-drugs/contact-based methods	29 (53.7)	7 (13.0)	3 (5.6)	15 (27.8)	0.173
Complementary and alternative therapies	20 (37.0)	18 (33.3)	1 (1.9)	15 (27.8)	0.013
Interventional methods	12 (22.6)	1 (1.9)	38 (71.7)	2 (3.8)	<0.001
Physical therapies	0 (0)	54 (96.4)	1 (1.9)	1 (1.9)	1.000
Group therapy	11 (20.4)	15 (27.8)	1 (1.9)	27 (50.0)	<0.001
Individual therapy	0 (0)	30 (55.6)	0 (0)	24 (44.4)	<0.001
Skills training	0 (0)	45 (80.4)	0 (0)	11 (19.6)	0.024
Pain education (for the patient)	0 (0)	50 (89.3)	0 (0)	6 (10.7)	0.651

The table presents the number (%) of participants who believed each pain assessment or management option was important or not important/neutral for understanding or helping the young person’s pain, dependent on condition (inflammatory or non-inflammatory). The first and second columns display concordant pairs (i.e. providing the same response for each vignette). The third and fourth columns display discordant pairs (i.e. providing a different response to each vignette). The *n* values and percentages exclude participants who stated they were ‘unfamiliar’ with individual pain management options. *P*-values were adjusted using the Holm–Bonferonni correction.

More than half of the pain management approaches were perceived to be a priority for only one condition, not both conditions. Three pharmacological pain management methods (NSAIDS, anti-rheumatic drugs, interventional methods) were perceived as important for the inflammatory condition but not the non-inflammatory condition. Four psychosocial therapies (group therapy, individual therapy, complementary and alternative therapies, skills training) and one biomedical method (drugs other than NSAIDS, anti-rheumatics or analgesics) were mostly important for the non-inflammatory condition, not the inflammatory condition.

### Implementing priorities in clinical practice

**Figure 1:**
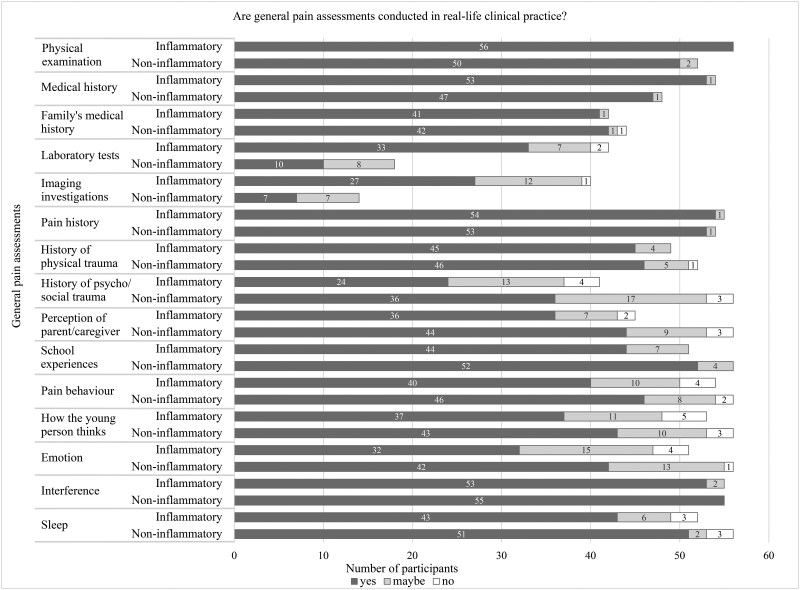
Number of participants who believed general pain assessments would be conducted in clinical practice. Bar chart showing participants’ responses (yes, no, maybe) to whether general pain assessment approaches would be conducted in clinical practice. The *n* values vary depending on the number of participants who rated each pain assessment approach as important for understanding the young person’s pain

**Figure 2:**
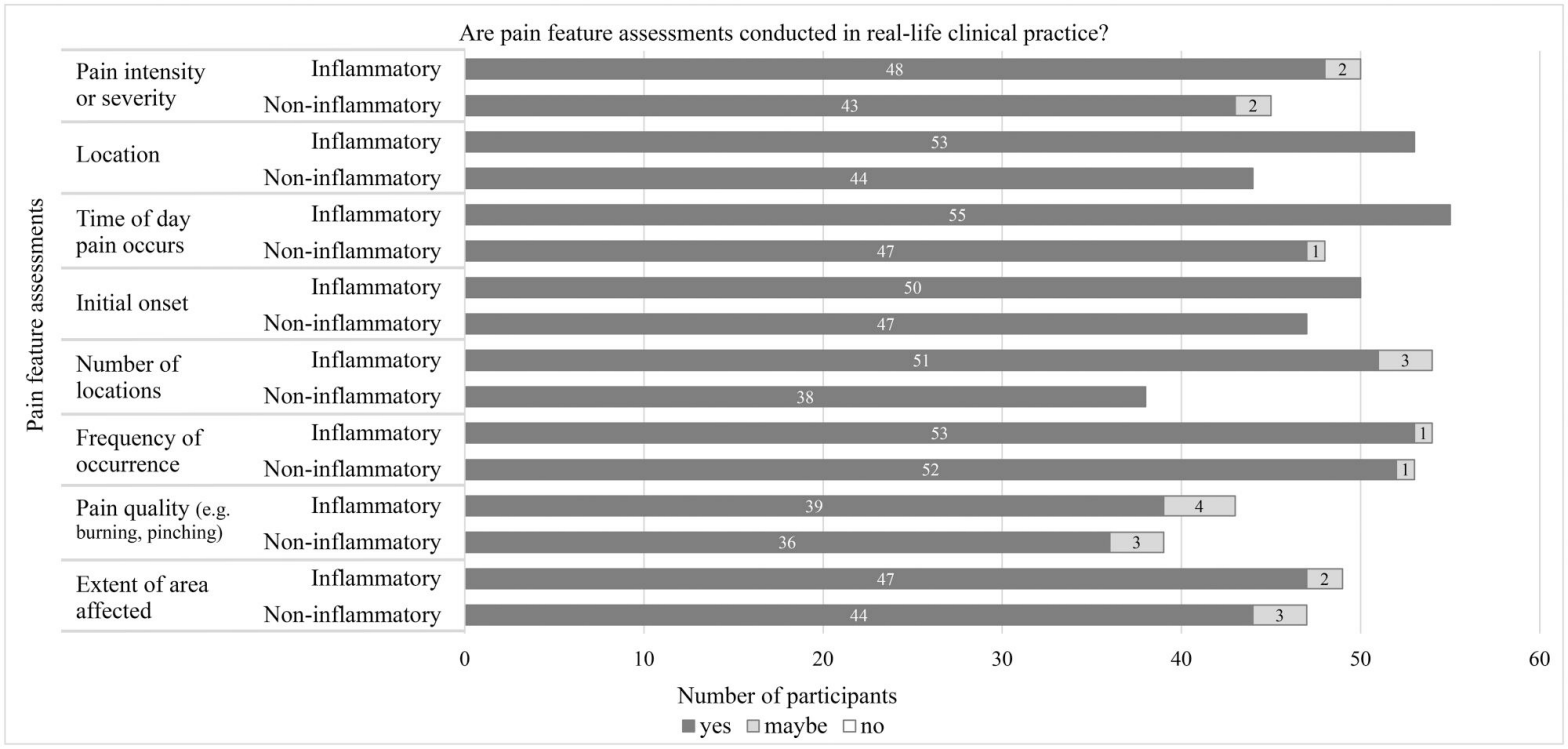
Number of participants who believed pain feature assessments would be conducted in clinical practice. Bar chart showing participants’ responses (yes, no, maybe) to whether pain feature assessment approaches would be conducted in clinical practice. The *n* values vary depending on the number of participants who rated each pain feature assessment approach as important for understanding the young person’s pain

**Figure 3:**
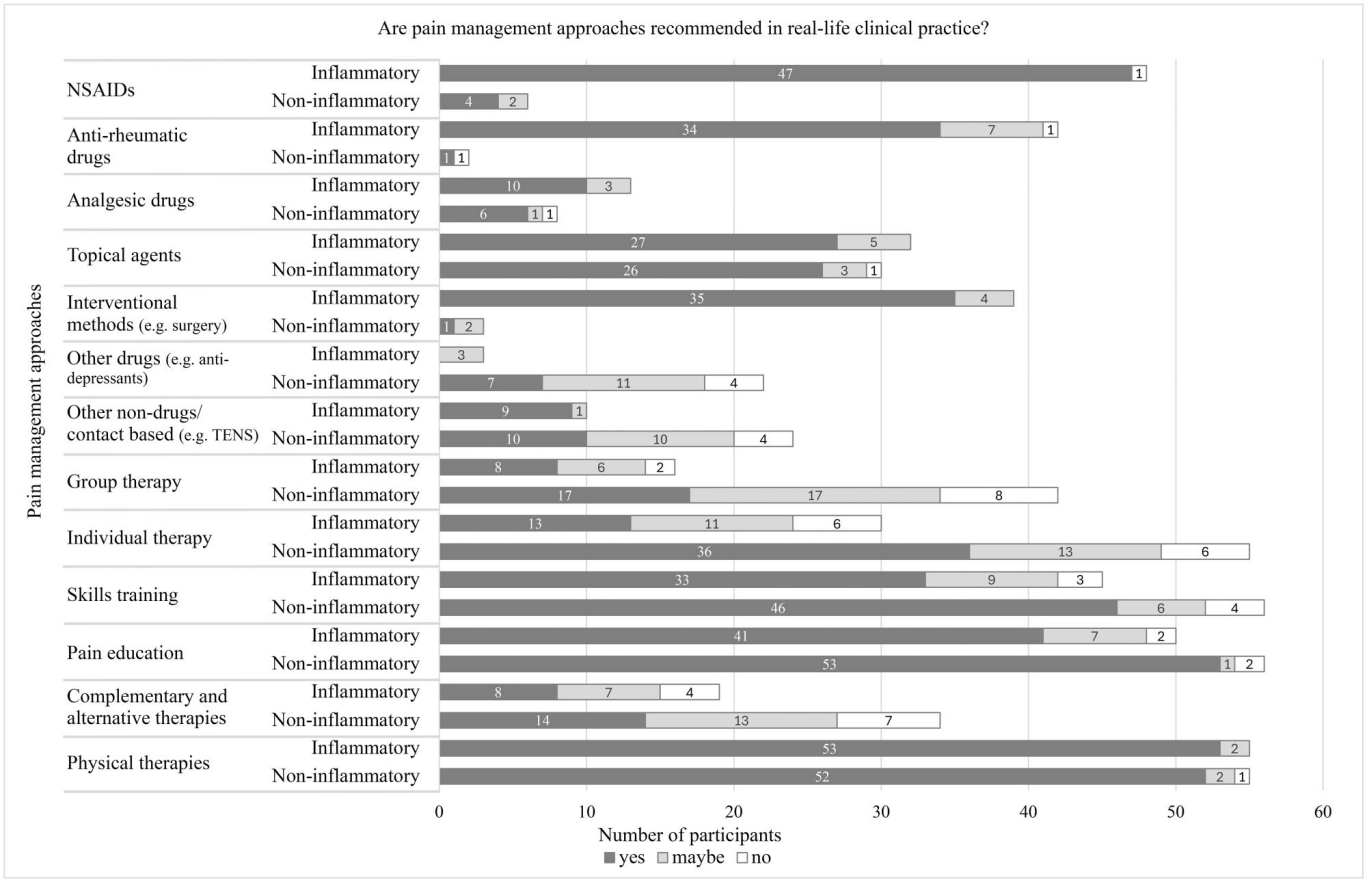
Number of participants who believed pain management approaches would be recommended in clinical practice. Bar chart showing participants’ responses (yes, no, maybe) to whether different pain management approaches would be conducted or recommended in clinical practice. The *n* values vary depending on the number of participants who rated each pain management approach as important for helping the young person’s pain

Participants did not believe that all general pain assessment (Fig. 1), pain feature assessment (Fig. 2) and pain management priorities (Fig. 3) would be implemented/recommended in clinical practice for both conditions. Examples include assessing history of psychosocial trauma or recommending talking therapies, such as CBT.

### Barriers to implementing priorities in clinical practice

Of the 56 participants, 54 provided responses about barriers to implementation of pain assessment and management priorities. Three categories of barriers were identified that were similar across conditions: clinical practical barriers, HCPs’ knowledge and skillset and HCPs’ beliefs about appropriate clinical care. Similarities and differences between conditions are discussed within the subcategories. Table 3 details the content analysis categories and subcategories, including the number of codes assigned to each subcategory and excerpts of participants’ responses.

**Table 3:** Content analysis of HCPs’ perceptions of barriers to implementation of pain assessment/management approaches.

Categories	Subcategories	Inflammatory		Non-inflammatory	
		Number of codes	Excerpts	Number of codes	Excerpts
1. Clinical practical barriers	1.1 Time restrictions	25	‘Time within clinic setting to explain pain’, P32, occupational therapist ‘Not always enough time to go into emotion/psychological background’, P27, paediatric/adolescent rheumatologist in training	10	‘Not always enough time to discuss emotional/trauma history in [a] busy general rheum clinic’, P27, rheumatologist in training ‘Time permitting to delve into potentially challenging subjects and issues’, P30, consultant paediatric/adolescent rheumatologist
	1.2 Resource availability	12	‘Lack of availability of psychology support is [the] biggest issue—[I] think [it] is very worthwhile but often very difficult to access’, P37, paediatric speciality trainee ‘Need a family therapist—not widely available’, P20, occupational therapist	30	‘Although psychological therapy is very important in the management of chronic pain, we do not have the resources to be able to refer every patient to the service’, P36, physiotherapist ‘Lack of resources, no referral pathways’, P52, physiotherapist
2. HCPs’ knowledge and skills	2.1 Limited pain assessment and/or management knowledge and skills	15	‘I am not skilled at assessing and addressing social and/or psych[ological] trauma’, P38, physiotherapist ‘I don’t know how to assess objectively emotion or coping strategies’, P26, consultant adult rheumatologist who sees adolescents	13	‘Don’t have the skills for acupuncture/ACT [acceptance and commitment therapy] in our team’, P32, occupational therapist ‘[The] non-pharmaceutical approach, [I’m] not as comfortable prescribing’, P53, paediatric specialist trainee
	2.2 Outside their professional remit	14	‘If I think that child couldn’t manage pain by herself/himself, and she/he is in a depressive mood, I consult the child to a professional working in psychology area’, P41, physiotherapist ‘As a physiotherapist, I am not able to order lab work or imaging’, P38, physiotherapist	18	‘These more sensitive questions would need to be explored—ideally by a psychologist if there is one in clinic’, P20, occupational therapist ‘Patient would be referred to the chronic pain service who would take over management of the pain’, P45, nurse
	2.3 Inability or ability to communicate with patients and families	7	‘I would wait until I have built a rapport with them to ask such questions’, P36, physiotherapist ‘Often patients [are] not forthcoming about trauma’, P33, consultant paediatric/adolescent rheumatologist	18	‘Uncertainty of providers how to approach certain questions’, P56, consultant rheumatologist ‘Reassuring/demonstrating ‘normal’ results to patient/family…I would discuss my expectations of the forthcoming results…and set them within the context of the clinical presentation (i.e. pain)’, P29, general paediatrician
3. HCPs’ beliefs about appropriate care	3.1 Biopsychosocial pain care restricted by habits and beliefs	38	‘If suspicion of inflammatory pain then medical management [is] relevant. If non-inflammatory pain, then a more physical, educational approach’, P29, general paediatrician ‘Typically [with] JIA the focus is more on the medical management and the functional impact rather than the psychosocial impact’, P31, physiotherapist	22	‘With no indication that there is a significant organic cause for the pain adding medications will feed into the sick role she is likely to adopt’, P22, physiotherapist ‘Need to be tailored depending on [the] patient[‘s] needs’, P6, consultant paediatric/adolescent rheumatologist

P: participant.

#### Category 1—Clinical practical barriers

##### Time restrictions

Participants similarly viewed their ability to conduct thorough psychosocial pain assessments and provide biopsychosocially informed pain management was impeded by time available during appointments and busy clinics across both inflammatory and non-inflammatory conditions.

##### Resource availability

In both conditions, access to pain assessment and management approaches was limited or no access was available, particularly for CYP with non-inflammatory conditions. Access issues predominantly related to psychosocial approaches (e.g. family therapy, psychology) for CYP with either condition. Participants described reprioritising patients ‘at risk of harm’ (P46), as a limited number of patients could be referred.

#### Category 2—HCPs’ knowledge and skills

##### Limited pain assessment and/or management knowledge and skills

Participants felt uncertain, uncomfortable or lacked confidence in their knowledge, experience or skills in implementing pain assessment and management for inflammatory and non-inflammatory conditions. Examples include lacking confidence in assessing psychosocial trauma or feeling uncertain about psychosocial therapies or alternative therapies (e.g. acceptance and commitment therapy, acupuncture) across both conditions.

##### Outside their professional remit

Participants could not conduct or recommend certain approaches for CYP with either inflammatory or non-inflammatory conditions, as these were limited to particular professional roles, such as prescribing medications or therapies (e.g. biologics, CBT). For those with non-inflammatory conditions, participants described requiring input from pain clinics to determine appropriate pain management. Some participants’ perceptions of their professional identity determined whether they believed it to be within their role or another person’s role to carry out certain approaches. This meant they referred patients to other services or specialists, particularly for psychosocial approaches, rather than carrying out brief interventions themselves. This included referring CYP with inflammatory or non-inflammatory conditions to psychologists to explore ‘sensitive questions’ (P20) or referring those with non-inflammatory conditions to chronic pain services.

##### Inability or ability to communicate with patients and families

For both conditions, participants’ ability to communicate with CYP and families acted as a barrier to provision of care. Pain communication depended on the health literacy of HCPs, CYP and families and/or the CYP’s or family’s willingness to engage. Engagement (e.g. with pain-focused management) was particularly emphasised as a barrier for those diagnosed with non-inflammatory musculoskeletal conditions that could be improved by HCPs communicating that there was no ‘biological’ cause (P13) for their pain, such as inflammation. Participants felt uncomfortable or uncertain with starting and facilitating conversations with patients with either condition about complex and abstract concepts, such as pain beliefs or psychosocial trauma, but particularly for those with non-inflammatory conditions. For both conditions, participants described having to be capable of determining whether CYP and families understood, believed and accepted the HCP’s decisions and pain management plans. This could be improved through building rapport and relationships with the family.

#### Category 3—HCPs’ beliefs about appropriate care

##### Biopsychosocial pain care restricted by habits and beliefs

Participants determined clinical pathways for pain assessment and management for CYP with inflammatory or non-inflammatory conditions by triaging by individual cases and contexts (e.g. patient’s demographics, examination findings). However, decisions were underpinned by personal habits and beliefs, particularly about the perceived cause of pain. Perceived cause included the diagnostic label (inflammatory or non-inflammatory musculoskeletal condition) and level of disease activity in inflammatory conditions (e.g. persistent pain alongside high disease levels versus persistent pain alongside low disease levels/no disease). Some HCPs restricted access to components of biopsychosocial pain assessment or management as they already perceived certain approaches would not be appropriate for a patient, depending on the perceived cause. A stepped approach was described for patients with inflammatory conditions that initially addressed physical causes of pain, such as inflammation, and prescribing medical or physical management (e.g. NSAIDs or injections). Management became more psychosocial (such as recommending pain education, CBT or other cognitive interventions for CYP) when HCPs’ beliefs shifted to perceive pain as non-inflammatory, i.e. if pain continued despite inflammation being controlled. In contrast, psychosocial pain management was predominantly prioritised for the non-inflammatory condition. Some participants perceived issues with using medication for patients with non-inflammatory conditions as it would not help their pain but could encourage CYP to adopt a ‘sick role’ (P22).

## Discussion

This is the first study to compare paediatric rheumatology HCPs’ pain assessment and management priorities and perceived barriers to implementing these in clinical practice for CYP with an inflammatory or non-inflammatory musculoskeletal condition. The findings are important as they provide new insights into how key decisions HCPs make for the provision of biopsychosocial pain care differ between conditions. Some HCPs prioritised biomedical pain management approaches (e.g. NSAIDs) for the inflammatory condition but not the non-inflammatory condition. Conversely, psychosocial approaches (e.g. talking therapies, skills training) were often not prioritised for the inflammatory condition but were considered important for managing pain in the non-inflammatory condition. Decisions depended on practical barriers, HCPs’ knowledge and skillset and their personal habits and beliefs. However, these decisions contrasted with clinical guidelines and paediatric chronic pain literature, which recommend implementing biopsychosocial pain assessment and management [8, 13, 17].

The study provides novel findings that while comprehensive pain assessments, essential for timely and effective pain management [8, 33], were viewed as important for both conditions, laboratory tests and imaging were not prioritised for the non-inflammatory condition. These tests are important for diagnosing and monitoring underlying causes of chronic pain (such as structural, inflammatory or infectious) but may not be routinely needed for non-inflammatory conditions unless symptoms change, suggesting a new underlying issue [7, 8, 12]. The vignette described a patient with a known diagnosis of chronic widespread pain, which may explain why these assessments were not prioritised. This has clinical implications for reducing unnecessary biomedical investigations in cases where a diagnosis is established and symptoms remain stable. Ideally throughout clinical practice, assessments of patient’s and family’s narratives and unique experiences of pain should be embedded in and used to inform HCPs’ decisions for personalised pain management plans [8]. Previous research suggests patients’ and families’ pain narratives are not always sought in paediatric rheumatology [10], but it was unclear which part of the narratives was overlooked. This study adds understanding by identifying that assessments of psychosocial trauma may not routinely occur for CYP with inflammatory or non-inflammatory musculoskeletal conditions. This gap has clinical relevance, as identifying psychosocial trauma, if present, can be key to developing effective, individualised pain management plans in both conditions [42].

There were notable differences in HCPs’ priorities between the two conditions regarding pain management approaches. It is important within clinical consultations that underlying disease be managed alongside addressing pain for inflammatory conditions [1, 16], yet previous research suggests psychosocial pain management is often deprioritised for CYP with inflammatory conditions [10]. This study is the first to identify specific psychosocial approaches often not prioritised for this group (e.g. group, individual and complementary therapies). Even when considered important, HCPs were unsure if these would be recommended in practice for either condition. The qualitative findings revealed that first-line treatments were disease focused, only moving to psychosocial approaches if pain persisted when disease was well-controlled. This has important clinical implications for patients with inflammatory musculoskeletal conditions. Patients may require support from HCPs to reconceptualise chronic pain in a biopsychosocial context. An example is if pain was communicated as an acute warning signal (e.g. inflammation) but persisted when disease was well-controlled [43]. CYP may subsequently lack an understanding of important pain self-management skills if these are not provided by healthcare teams. These skills help improve CYPs’ health and pain outcomes [21, 42, 44] and CYP deem them key for successful transition into adult services and independence [45]. Therefore HCPs should ideally incorporate and communicate the psychosocial context of pain early in and throughout clinical care to help CYP conceptualise pain as biopsychosocial.

For the non-inflammatory condition, HCPs’ focus on psychosocial aspects of pain, like recommending talking therapies, may lead CYP to perceive their pain is dismissed or invalidated. Sometimes unintentionally, HCPs can imply pain is exaggerated [24] or associated with a mental health condition [46]. Perceived pain dismissal can lead CYP to self-blame for having chronic pain and/or become less engaged in clinical care and treatment of their condition [25, 46]. In complex chronic pain, CYP and parents ‘buy in’ or assign credibility to physical causes of pain (inflammation) over non-physical explanations (idiopathic pain) [47]. HCPs in this study identified this as a barrier to involving families in pain management. This highlights the clinical value of HCPs empathically communicating about pain and addressing misconceptions about chronic pain with families [43]. This can reduce perceived pain dismissal by providing time and commitment to explore and manage the cognitive and emotional impact of pain [24, 43, 48].

This study extends knowledge by identifying that common barriers affect HCPs’ ability to implement and achieve biopsychosocial care across both musculoskeletal conditions, which had not previously been established. These include a lack of confidence, knowledge and/or skills to effectively assess, manage and communicate about pain. This barrier is common in other paediatric chronic pain conditions [9, 10, 26] and is expected given the limited, high-quality pain training or education resources available to HCPs [9, 49]. Limited time and resources also impeded their ability to provide thorough psychosocial pain assessment and management, an issue prevalent in paediatric pain management [10, 27]. Importantly, HCPs referred patients to other services or specialists, such as pain services or psychologists, rather than conducting brief assessments and interventions themselves. This highlights a critical opportunity in the clinical pathway to deliver brief assessments. An example of such an opportunity includes patients completing a brief psychosocial pain profile prior to clinics to allow HCPs to prioritise their clinic time to address CYP’s or parents’ concerns.

Future research should explore pain education for paediatric rheumatology HCPs, particularly models of pain that address causal pain beliefs by demonstrating the interconnectedness of physiological and psychological contributions of pain. This is important given that pain management decisions varied according to these beliefs. Education targeting causal pain beliefs may support informed decision-making and promote a holistic approach to pain assessment and management. Future research should also explore training to assist HCPs in assessing and discussing pain with CYP to reduce organisational constraints on their decision-making. An example includes motivational interview training which can save time and resources and potentially be preventative in that HCPs set patient’s expectations and beliefs early in clinical care [50].

A strength of this study is that it captured HCPs’ perspectives on perceived ideal practice for pain assessment/management compared with what they believed could be implemented in clinical practice. This revealed nuances in HCPs’ decisions, highlighting that they recognise the importance of psychological services for both conditions but face limited access to providing these resources to CYP in real-world clinical contexts. HCPs were not asked if assessment and management approaches they deemed ‘unimportant’ would be conducted in clinical practice to reduce questionnaire burden and prevent participant fatigue, which could affect data quality. This limits findings, as it is unclear whether certain approaches may be used despite being rated unimportant. A limitation of this study is the underrepresentation of some multidisciplinary HCPs, particularly those assumed to have received more training in biopsychosocial care (e.g. occupational therapists, psychologists). However, pain content in training curricula is often limited even within these disciplines [9]. Additionally, few participants had large non-inflammatory clinical workloads (e.g. in specialist pain services). While ideally all HCPs are recommended to manage pain biopsychosocially [8, 33], underrepresented groups in this study may hold different perspectives and priorities than those with more medical training (e.g. doctors, nurses) or predominantly inflammatory clinical workloads. Participants were recruited internationally to gain a global perspective on pain assessment/management. However, more than half of the participants resided in the UK, which could potentially limit conclusions that can be drawn on the perspectives of health professionals internationally. This likely occurred because of the recruitment strategy. Advertisements were distributed through multiple UK-based clinical and professional organisations but only via social media and an e-mail list server for international participants. Advertising through clinical and professional organisations is an efficient recruitment strategy that enables researchers to target specific audiences. Key differences in opinions are likely present between countries (e.g. prioritising pharmacological versus psychosocial management approaches) due to differences in training and clinical standards and in clinical contexts and infrastructures across countries.

In conclusion, this study provides evidence that paediatric rheumatology HCPs’ decisions towards the prioritisation of pain assessment and management differ between inflammatory and non-inflammatory paediatric chronic musculoskeletal conditions. Psychosocial pain management approaches were prioritised for those with a non-inflammatory condition whereas initial pain treatment was mostly disease-focused for inflammatory conditions. Ideally an integrated biopsychosocial approach should be provided irrespective of condition type, as chronic pain experiences are the result of complex interactions between biological, psychological and social factors. To support this, future research should explore pain education for paediatric rheumatology HCPs, with particular attention to the biopsychosocial contributions to pain.

## Supplementary Material

rkag007_Supplementary_Data

## Data Availability

The data underlying this article will be shared upon reasonable request to the corresponding author.
