# Supplementary material for: Biopsychosocial pain assessment and management in paediatric inflammatory vs non-inflammatory musculoskeletal conditions: a vignette study
Source: Rheumatol Adv Pract. 2026 Jan 19;10(1):rkag007. doi: 10.1093/rap/rkag007 (PMC12937585; doi:10.1093/rap/rkag007)
Supplement: rkag007_Supplementary_Data [file rkag007_supplementary_data.zip › Supplementary_Data_S1.docx]

**Supplementary Data S1:** Fictional vignettes

| **Vignette type** | **Contents of the vignette** |
| --- | --- |
| **Inflammatory** | “You are in your specialist paediatric rheumatology clinic on a Thursday and a 13-year old [girl/boy] is attending for a follow-up. [He/She] was diagnosed with Juvenile Idiopathic Arthritis (JIA) 6-months ago. [She/He] reports that [she’s/he’s] been experiencing regular musculoskeletal pain in both knees for more than 3 months and, when asked, [she/he] scores the pain intensity as a 6 out of 10. Upon examination, you note swelling and restricted range in only one knee.” |
| **Non-inflammatory** | “On a Tuesday, you are in your specialist paediatric rheumatology clinic and a 13-year old [boy/girl] attends for a follow-up in relation to their condition, diffuse idiopathic pain (i.e., chronic widespread pain/juvenile fibromyalgia), which was diagnosed 6-months earlier. [He/She] reports that [he’s/she’s] been experiencing regular diffuse musculoskeletal pain for more than 3 months and when rating [her/his] pain intensity out of 10 [he/she] gives it a score of 6. Upon examination, your findings are consistent with the diagnosis, including signs of allodynia and no joint swelling.” |
