# Supplementary material for: Biopsychosocial pain assessment and management in paediatric inflammatory vs non-inflammatory musculoskeletal conditions: a vignette study
Source: Rheumatol Adv Pract. 2026 Jan 19;10(1):rkag007. doi: 10.1093/rap/rkag007 (PMC12937585; doi:10.1093/rap/rkag007)
Supplement: rkag007_Supplementary_Data [file rkag007_supplementary_data.zip › Supplementary_Data_S2.docx]

**Supplementary Data S2. Questionnaire**

| **Section** | **Questions** |
| --- | --- |
| Demographics | 1. What is your gender?  - Male - Female - Non-binary - Prefer to self-describe (provides text box) - Prefer not to say  1. What is your current job title?  - Consultant paediatric or adolescent rheumatologist - Consultant adult rheumatologist who sees adolescents - Paediatric or adolescent rheumatologist in training - General Paediatrician with an interest in paediatric rheumatology - Occupational therapist in Paediatric or Adolescent Rheumatology - Physiotherapist in Paediatric or Adolescent Rheumatology - Nurse in Paediatric or Adolescent Rheumatology - Psychologist - Paediatric speciality trainee (rheumatology) - Pain specialist doctor - Other (please specify)  1. Please state how many years you have been registered as a health professional.  - Drop down of number of years (less than 1 year up to 24, then more than or equal to 25 years)  1. Please state how many years you have worked in the paediatric/adolescent field  - Drop down of number of years (less than 1 year up to 24, then more than or equal to 25 years)  1. In which country are you currently working?  - Drop down list of countries |
| **Vignette scenario number one presented** | |
| Pain assessment | 1. Thinking about how this [girl/boy] might be managed in clinic, please rate how important you believe the following assessments are for understanding [her/his] pain. (Acknowledging that some of these assessments may have been performed/documented at previous appointments and/or by other team members)   (5 point Likert: not at all important, low importance, neutral, important, very important)   - Physical examination (e.g., range of motion, posture) - Medical history - Family's medical history - Laboratory tests (e.g., bloods) - Imaging investigations (e.g., x-ray, MRI) - Pain history (e.g., when it started) - History of physical trauma - History of psychological and/or social trauma - Perception of their parent(s)/caregiver(s) - School experience(s) (e.g., attendance, engagement, past/present) - Pain behaviour (e.g., young person’s coping strategies) - How the young person thinks (beliefs about or focus on pain) - Emotion - Interference (e.g., daily activities, hobbies, sports) - Sleep  1. “From the pain assessments that you stated are 'very important' or 'important', please indicate in your opinion whether these would be assessed in your real-world clinical practice.”  - Provides a list of the options the participant selected ‘very important’ or ‘important’ for and they select - Yes/no/maybe - If they tick ‘no’ or ‘maybe’ to any of the options then another question presents:  1. “In the previous question, you selected either 'maybe' and/or 'no' for one or more options. Please advise why you do not think these pain assessment(s) would be conducted.” 2. “Please rate how important you believe it is to examine the below pain features for this [girl/boy].”    - (5-point Likert scale: Not at all important – Very important)      - Pain intensity or severity      - Location      - Time of day pain occurs      - Initial onset      - Number of locations      - Frequency of occurrence      - Quality (e.g., burning, pinching)      - Extent of area affected 3. “From the pain features that you stated are 'very important' or 'important', please indicate in your opinion whether these would be assessed in your real-world clinical practice.”    - Yes/no/maybe    - If they tick ‘no’ or ‘maybe’ to any of the options then another question presents: 4. “In the previous question, you selected either 'maybe' and/or 'no' for one or more options. Please advise why you do not think these pain feature(s) would be assessed?” |
| Pain management | 1. “From the list below, please rate how important you believe each management option is for helping [her/his] pain.”   (5 point Likert: Very important, important, neutral, low importance, not at all important, and the option for ‘Unfamiliar with method’)   - Nonsteroidal anti-inflammatory drugs (NSAIDS) - Anti-rheumatic drugs (e.g., DMARDS, biologics) - Analgesic drugs (e.g., opioid or non-opioid) - Topical agents (e.g., deep heat, heat/ice) - Interventional methods (e.g., surgery, nerve block, local injection therapy, intra-articular joint injections) - Other drugs (e.g., antidepressants, anticonvulsants) - Other non-drugs/contact based (e.g., transcutaneous electrical nerve stimulation (TENS), acupuncture) - Group therapy (e.g., family therapy) - Individual therapy (e.g., cognitive behavioural therapy, acceptance and commitment therapy) - Skills training (e.g., coping strategies, stress management, mindfulness, sleep hygiene, relaxation) - Pain education (e.g., explain pain) - Complementary and alternative therapies (e.g., music therapy, hypnotherapy, biofeedback) - Physical (e.g., exercise, hydrotherapy, massage)  1. “From the pain management options that you stated are 'very important' or 'important', please indicate whether you believe that these would likely be recommended in your real-world clinical practice.”  - Yes/no/maybe - If they select no or maybe to any of the options then another question presents:  1. “In the previous question, you indicated either 'maybe' and/or 'no' for one or more options. Please advise why you believe these pain treatments would not be recommended” |
| **Vignette scenario number two presented and the same questions are asked** | |
| Final questions | 1. Thinking about the children/young people you see in an average week, what percentage have inflammatory musculoskeletal conditions? (e.g., juvenile idiopathic arthritis, systemic lupus erythematosus)  - Drop down options: less than 25%, 25% - 50%, 50% - 75%, over 75%  1. Thinking about the children/young people you see in an average week, what percentage have non-inflammatory musculoskeletal conditions with a known cause? (e.g., hypermobility)    - Drop down options: less than 25%, 25% - 50%, 50% - 75%, over 75% 2. Thinking about the children/young people you see in an average week, what percentage have non-inflammatory musculoskeletal conditions without a known cause? (e.g., diffuse idiopathic pain)    - Drop down options: less than 25%, 25% - 50%, 50% - 75%, over 75% 3. On a scale of 0-100, how confident are you in your understanding of pain? (0 being not at all confident and 100 being confident) |
